# Study on the correlation between abnormal bone metabolism and cognitive impairment in type 2 diabetes mellitus

**DOI:** 10.3389/fmed.2025.1530462

**Published:** 2025-05-09

**Authors:** Jiang Li, Yuxiao An, Jian Qin, Noor Shafini Mohamad, Izzad Ramli

**Affiliations:** ^1^Faculty of Health Sciences, Universiti Teknologi MARA, Shah Alam, Malaysia; ^2^The Second Affiliated Hospital of Shandong First Medical University, Taian, China; ^3^Medical Imaging, Faculty of Health and Life Sciences, Exeter, United Kingdom; ^4^College of Computing, Informatics and Mathematics, Universiti Teknologi MARA, Shah Alam, Malaysia

**Keywords:** type 2 diabetes mellitus, abnormal bone metabolism, cognitive impairment, bone mineral density, osteocalcin

## Abstract

**Introduction:**

Type 2 diabetes mellitus (T2DM) is often accompanied by bone metabolic disorders and cognitive impairment, forming an interactive network through metabolic derangements, oxidative stress, and inflammatory responses. Hyperglycemia and insulin resistance disrupt bone remodeling leading to osteoporosis while simultaneously impairing cognition via blood-brain barrier damage and neuroinflammation. Osteogenic factors like osteocalcin may bidirectionally regulate glucose metabolism and brain function, suggesting that “bone-brain axis” dysregulation could be a potential mechanism underlying cognitive impairment in T2DM. This study aims to characterize cognitive function patterns in T2DM patients with bone metabolic abnormalities and their clinical correlations, providing a basis for multisystemic interventions.

**Methods:**

The general clinical data, osteocalcin (OC), glycosylated hemoglobin (HbA1c), bone mineral density (BMD), and the Montreal Cognitive Assessment (MoCA) scores of 50 patients with T2DM were collected. According to whether cognitive impairment occurred or not, one-way ANOVA was performed to analyze the correlation between cognitive and clinical indicators, BMD and OC. Multiple linear regression analysis was performed with cognition and bone density as dependent variables and other factors as independent variables.

**Results:**

T2DM subjects were grouped according to bone mass. The osteoporosis group had the lowest MoCA score and bone density, followed by the osteopenia group. There were 16 cases (16/17 94.12%) of cognitive impairment in the osteoporosis group, 13 cases (13/17 76.47%) of cognitive impairment in the osteopenia group, and 3 cases (3/16 18.75%) of cognitive impairment in the normal bone mass group. Compared with the normal cognitive group, the MoCA score, OC measurement and BMD of the patients in the cognitive impairment group were lower (*P* < 0.05). BMD (*r* = 0.686, *P* = 0.000), OC (*r* = 0.756, *P* = 0.000) are positively correlated with MoCA score. OC (*r* = 0.690, *P* = 0.000) and Age (*r* = −0.032, *P* = 0.045) are positively correlated with BMD. Multivariate linear regression analysis found that with cognition as the dependent variable, the decrease in BMD (*P* = 0.028) and OC (*P* = 0.000) aggravated the occurrence of cognitive impairment; with BMD as the dependent variable, the decline in cognition (*P* = 0.028) and OC (*P* = 0.029) aggravated the decrease in BMD.

**Conclusion:**

T2DM, osteoporosis, and cognitive impairment form pathological connections through metabolic disorders, chronic inflammation, and bidirectional regulatory networks of the “bone-brain axis,” with osteocalcin serving as a key mediator that maintains bone remodeling balance while also exerting cross-domain regulation over central insulin signaling and synaptic plasticity. Understanding these interactive mechanisms provides a basis for developing combined screening models integrating bone density and cognitive assessments, and promotes multidisciplinary collaborative interventions across endocrinology, orthopedics, and neurology to improve overall outcomes for T2DM patients.

## 1 Introduction

Diabetes mellitus is a chronic metabolic disorder affecting a significant global population. According to projections by the International Diabetes Federation, the global prevalence of diabetes is expected to reach 642 million by 2040 (1). Type 2 diabetes mellitus (T2DM), the most common form of diabetes, is characterized by insulin resistance and chronic hyperglycemia, with pathological processes involving multiple organ systems including microcirculation, retina, kidneys, peripheral nerves, bone metabolism, and cognitive function (2–4). Emerging evidence has highlighted the comorbidity of T2DM with bone metabolic disorders and cognitive dysfunction in recent years. Studies have demonstrated that T2DM patients exhibit impaired bone remodeling processes (5), increased fracture risk (6), and significantly elevated incidence of cognitive impairment (7, 8), with a substantial proportion progressing to dementia (9). This intricate relationship suggests that T2DM, bone metabolism, and cognitive dysfunction may form a pathological cascade network through metabolic perturbations, inflammatory responses, and neuroendocrine interactions.

The pathophysiological mechanisms underlying bone metabolic abnormalities in T2DM patients involve multiple factors (10): (1) Deposition of advanced glycation end products (AGEs) in bone matrix directly impairs bone biomechanical properties; (2) Insulin resistance and hyperglycemia disrupt osteogenic differentiation and accelerate bone resorption through interfering with multiple signaling pathways; (3) Aberrant secretion of bone turnover markers such as osteocalcin (OC) further disturbs the dynamic balance of bone remodeling. Clinical evidence demonstrates characteristic alterations of reduced cortical bone density and increased bone fragility in T2DM patients (11). Furthermore, obesity and visceral adipose tissue deposition accelerate bone loss through releasing inflammatory cytokines (e.g., IL-6, TNF-α) that activate osteoclast activity (12), suggesting metabolic inflammation serves as a critical bridge linking T2DM and osteoporosis.

Chronic hyperglycemia can impair the central nervous system through multiple pathways (13–15), including insulin signaling dysfunction, AGEs-induced oxidative stress and neuroinflammation, and alterations in blood-brain barrier permeability. Notably, a bidirectional interaction exists between bone metabolism and cognitive function. A cross-sectional study in obese individuals (16) revealed that low serum OC levels are associated with cognitive decline and alterations in brain microstructure. Furthermore, reduced bone density is significantly correlated with increased risk of Alzheimer’s disease (AD), suggesting that dysregulation of the bone-brain axis may serve as a critical mediating mechanism for cognitive impairment in T2DM patients.

Although previous studies have separately investigated the associations among T2DM, bone metabolic abnormalities, and cognitive impairment, the interactive mechanisms linking these three components remain incompletely elucidated. Whether bone metabolic abnormalities independently mediate cognitive decline in T2DM patients beyond glycemic metabolic disorders requires further clarification. Additionally, the dual regulatory mechanisms of bone-derived factors in glucose metabolism and central nervous system modulation remain unresolved. This study focuses on T2DM patients with concurrent bone metabolic abnormalities, aiming to clarify cognitive function disparities across different bone mass subgroups and their association patterns with clinical parameters. The findings will provide a theoretical foundation for early cognitive screening and interdisciplinary interventions in this high-risk population.

## 2 Materials and methods

### 2.1 Subjects

This study included 50 subjects with T2DM (age range: 45–69 years old, 25 women and 25 men) who were treated in the Second Affiliated Hospital of Shandong First Medical University from July 2023 to December 2023. Inclusion criteria: (1) Fasting blood glucose ≥ 7.0 mmol/L, OGTT 2-h blood glucose ≥ 11.0 mmol/L. (2) Glycated hemoglobin (HbA1c) > 7%. (3) No insulin is used, only hypoglycemic drugs are used to control blood sugar. (4) None Diabetic nephropathy, diabetic eye disease, diabetic foot and other complications. (5) No hypertension. (6) Educational years > 6 years. Subjects should be required to meet all the above conditions. Exclusion criteria: (1) People with acute complications of diabetes, acute inflammation, severe liver and kidney insufficiency, cancer, type 1 diabetes and other special types of diabetes. (2) Conditions affecting bone metabolism, including hyperthyroidism, hypercortisolism, connective tissue diseases, and glucocorticoid use, are excluded. (3) People with cerebral infarction and severe demyelinating lesions on magnetic resonance examination. (4) People with metal implants and claustrophobia. (5) People with a history of mental illness. (6) People with a recent history of mental illness; Have used bisphosphonates, calcium, vitamin D and other osteoporosis drugs in the past 3 months. (7) Subjects with a history of hormonal drug use or any of the above conditions would be excluded.

### 2.2 General information

Basic information such as the patient’s age, gender, height, weight, and years of education were collected, and the body mass index (BMI) was calculated based on height and weight. Venous blood was drawn from all enrolled patients at 6:30 the next morning after fasting for 10 h, and fasting blood glucose, 2-h postprandial blood glucose, and serum OC were measured using a fully automatic biochemical analyzer. Determination of HbA1c by high-performance liquid chromatography.

### 2.3 Bone mineral density measurement

Dual-energy X-ray absorptiometry (Horizon W, Hologic Inc., United States) was used to detect the bone mineral density (BMD) of the subjects’ lumbar vertebrae in the anteroposterior position (L1–L4). T2DM patients were divided into 3 groups using the T score method recommended by WHO (17): Normal bone mass group: T value ≥ –01.0, osteopenia group: −1 > T > −2.5, osteoporosis group: T ≤ −2.5 indicates osteoporosis.

### 2.4 MRI examination

Image data were collected using a GE 3.0T magnetic resonance scanner (Discovery MR750, General Electric, Milwaukee, WI, United States), combined with an eight-channel coil for the head and neck. The subject lies flat on the examination bed, with the head pillowed in the head examination coil. The scanning positioning line is placed at the midpoint of the line between the subject’s eyebrows. Noise-reducing earplugs are used to isolate the noise. Routine scanning of T1WI, T2WI, T2WIFLAIR, and DWI sequences has eliminated intracerebral lesions that would affect the research results.

### 2.5 Cognitive assessment

The Montreal Cognitive Assessment (MoCA) was used to evaluate the patient’s cognitive ability. The total MoCA score is 30 points. The score standard for mild cognitive impairment is MoCA < 26 points. Since the MoCA score is significantly affected by education level, the score of subjects with less than 12 years of education will be additional one cent. MoCA testing is performed in a quiet room by trained professionals.

### 2.6 Statistical analysis

Statistical analysis was performed using SPSS Statistics version 23.0. Measurement data were expressed as ($\bar{\text{x}}$ ± s). When the data were normally distributed, independent samples *t*-test was used for comparisons between two groups, and pairwise comparisons were conducted using the least significant difference (LSD) method. When the data is not normally distributed, the rank sum test is used. Count data were analyzed using *Chi-square* test or Fisher’s exact test. The correlation between BMD, MoCA and other data was analyzed by Spearman rank correlation analysis. Multiple linear stepwise regression analysis with MoCA and BMD as dependent variables and other factors as independent variables. *P* < 0.05 means the difference is statistically significant.

## 3 Results

### 3.1 Comparison of clinical data between different bone mass groups

All subjects were divided into the osteoporosis group (*n* = 17), osteopenia group (*n* = 17), and control group (*n* = 16) according to the T value. There were statistically significant differences in MoCA score, OC, and BMD between the three groups of patients (*p* < 0.05). Through comparison, it can be found that the MoCA, OC, and BMI of the osteoporosis group were significantly lower than those of the other two groups, and the control group had the highest values. There was no significant difference in age, gender, weight, height, BMI, years of education, and HbA1c among the three groups of patients (*p* > 0.05) (Table 1).

**TABLE 1:** Subjects statistical table of clinical data between different bone mass groups.

	Osteoporosis group (I) (*n* = 17)	Osteopenia group (II) (*n* = 17)	Control group(III) (*n* = 16)	*F*	*p-*value
Age	58.76 ± 6.09	61.00 ± 5.17	56.56 ± 4.55	2.867	0.067
Gender (male/female)	7/10	10/7	8/8	0.508	0.605
Weight (Kg)	71.18 ± 11.71	72.59 ± 11.68	72.06 ± 10.16	0.069	0.934
Height (cm)	166.35 ± 7.34	164.47 ± 7.92	165.94 ± 6.71	0.307	0.737
BMI (Kg/m^2^)	25.65 ± 3.48	26.84 ± 4.02	26.17 ± 3.31	0.462	0.633
Education (years)	10.94 ± 3.73	9.82 ± 2.13	12.50 ± 3.46	2.937	0.063
MoCA	21.88 ± 2.69	23.82 ± 2.40	27.00 ± 1.63	20.746	0.000*
HbA1c	8.99 ± 2.07	8.69 ± 1.64	8.42 ± 1.71	0.413	0.664
OC (ng/mL)	23.13 ± 2.58	25.28 ± 2.95	28.73 ± 3.58	14.064	0.000*
BMD (g/cm^2^)	0.75 ± 0.09	0.94 ± 0.07	1.09 ± 0.06	90.464	0.000*

*Indicates that the difference between groups is statistically significant (*p* < 0.05).

### 3.2 Analysis of differences between groups based on cognitive changes

According to whether cognitive impairment occurred, all subjects were divided into a cognitive impairment group (*n* = 32) and a normal cognitive group (*n* = 18). There were no statistically significant differences in age, weight, height, BMI, HbA1c, and years of education between the groups (*p* > 0.05). Compared with the normal cognitive group, the MoCA, OC, BMD, and T values of patients in the cognitive impairment group were significantly lower than those in the normal cognitive group, and the differences were statistically significant (*p* < 0.05). The comparison results are shown in Table 2.

**TABLE 2:** Statistical table of differences between groups in cognitive changes.

	Cognitive impairment group (*n* = 32)	Cognitive normal group (*n* = 18)	*t*	*p*-value
Age (years)	59.56 ± 5.75	57.50 ± 4.97	1.276	0.208
Weight (Kg)	71.59 ± 11.86	72.56 ± 9.62	−0.294	0.770
Height (cm)	165.78 ± 7.49	165.22 ± 6.99	0.259	0.796
BMI (Kg/m^2^)	26.01 ± 3.80	26.59 ± 3.22	−0.540	0.592
HbA1c	8.841 ± 1.94	8.47 ± 1.55	0.692	0.492
Education (years)	10.59 ± 3.14	11.89 ± 3.51	−1.341	0.186
MoCA	22.28 ± 2.04	27.56 ± 1.04	−10.232	0.000*
OC (ng/mL)	23.84 ± 2.30	28.88 ± 3.79	−5.865	0.000*
BMD (g/cm^2^)	0.86 ± 0.14	1.04 ± 0.11	−4.580	0.000*
*T*-value	−2.42 ± 0.82	−0.24 ± 1.03	−8.198	0.000*

*Indicates that the difference between groups is statistically significant (*p* < 0.05).

### 3.3 Correlation analysis between Montreal Cognitive Assessment score and clinical parameters

**FIGURE 1:**
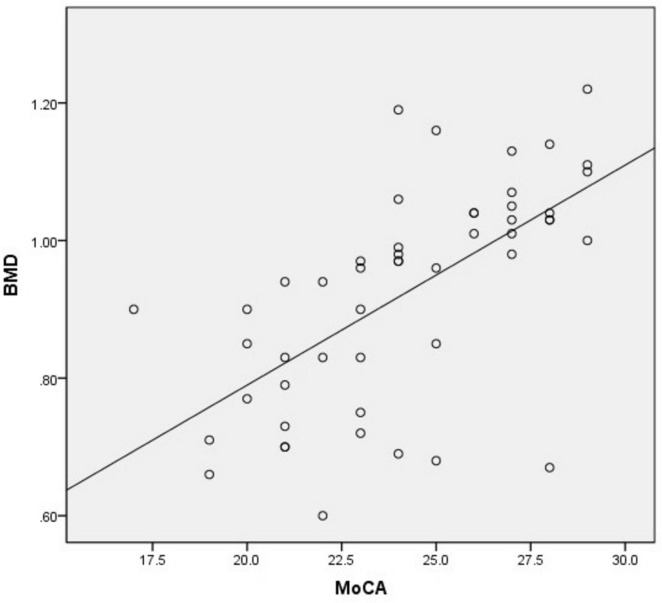
Linear model of the relationship between BMD and MoCA (*r* = 0.686, *P* = 0.000, *R*^2^ = 0.389).

**FIGURE 2:**
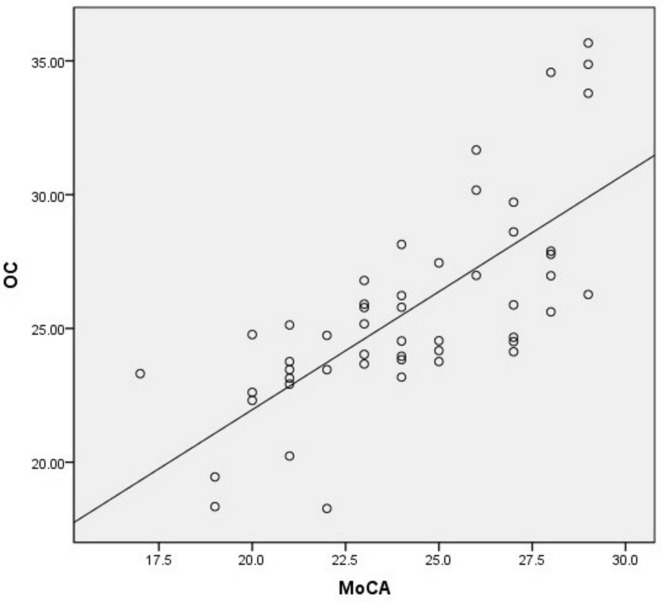
Linear model of the relationship between OC and MoCA (*r* = 0.756, *P* = 0.000, *R*^2^ = 0.520).

BMD (*r* = 0.686, *p* = 0.000), OC (*r* = 0.756, *p* = 0.000) are positively correlated with MoCA score, *p* < 0.05. HbA1c (*r* = −0.032, *p* = 0.825), age(*r* = −0.190, *p* = 0.185) has no correlation with the MoCA score (Table 3 and Figures 1, 2).

**TABLE 3:** Spearman correlation analysis between MoCA score and multiple indicators.

	*r*	*p*
BMD	0.686	0.000*
OC	0.756	0.000*
HbA1c	0.032	0.825
Age (years)	−0.190	0.185

*Indicates that the difference between groups is statistically significant (*p* < 0.05).

### 3.4 Correlation analysis between BMD score and clinical parameters

**FIGURE 3:**
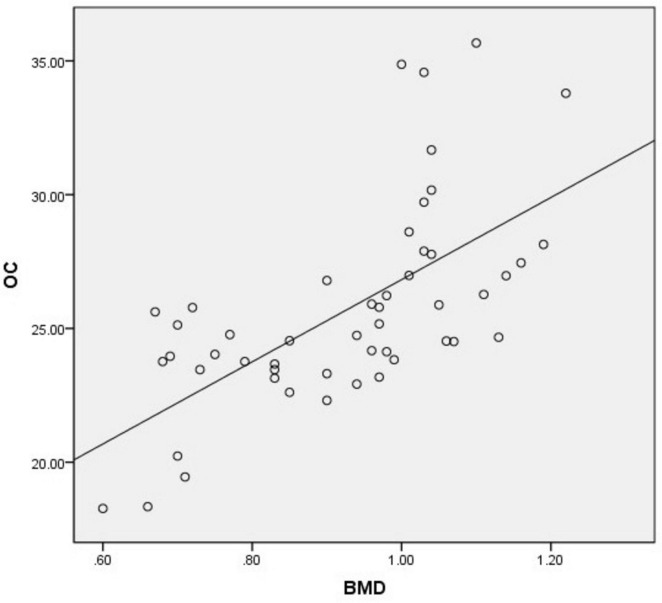
Linear model of the relationship between OC and BMD (*r* = 0.690, *P* = 0.000, *R*^2^ = 0.414).

**FIGURE 4:**
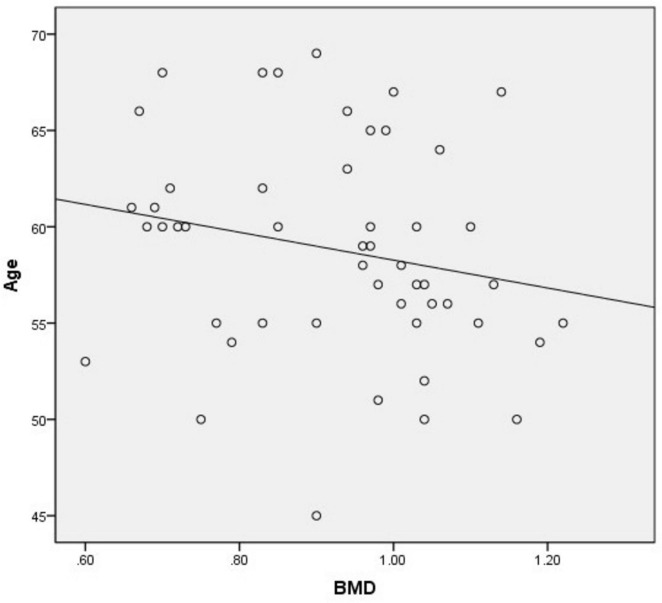
Linear model of the relationship between age and BMD (*r* = - 0.032, *P* = 0.045, *R*^2^ = 0.043).

There was a significant positive correlation between OC and BMD (*r* = 0.690, *p* = 0.000). There was a weak positive correlation between age and BMD (*r* = −0.032, *p* = 0.045). There was no significant correlation between HbA1c (*r* = −0.032, *p* = 0.825), gender (*r* = 0.028, *p* = 0.848), BMI (*r* = −0.124, *p* = 0.392) and BMD (Table 4 and Figures 3, 4).

**TABLE 4:** Spearman correlation analysis between BMD score and clinical parameters.

	*r*	*p*
OC	0.690	0.000*
HbA1c	−0.032	0.825
Gender	0.028	0.848
Age (years)	−0.028	0.045*
BMI	0.124	0.392

*Indicates that the difference between groups is statistically significant (*p* < 0.05).

### 3.5 Multiple linear regression analysis

Multiple linear regression analysis was performed with MoCA as the dependent variable and other factors as independent variables (Table 5). The significance test results showed that the reduction of BMD and OC aggravated the occurrence of cognitive impairment (*p* < 0.05).

**TABLE 5:** Results of multiple linear regression analysis with MoCA as the dependent variable.

	B	Standard error	Standardized β	*t*	*p*
BMD	6.020	2.647	0.309	2.274	0.028*
OC	0.430	0.108	0.527	3.998	0.000*
HbA1c	0.124	0.176	0.072	0.708	0.483
Age	−0.004	0.064	−0.007	−0.062	0.951
Weight	−0.308	0.443	−1.097	−0.694	0.492
Height	−0.230	0.400	0.540	0.575	0.568
BMI	0.772	1.198	0.895	0.645	0.523

*Indicates that the difference between groups is statistically significant (*p* < 0.05). Multiple linear regression analysis was performed with BMD as the dependent variable and other factors as independent variables. The results are shown in Table 6. Significance test results show that the decline in cognition and OC aggravates the decline in BMD (*p* < 0.05).

**TABLE 6:** Results of multiple linear regression analysis with BMD as the dependent variable.

	B	Standard error	Standardized β	*t*	*P*
MoCA	0.018	0.008	0.355	2.274	0.028*
OC	0.015	0.007	0.354	2.259	0.029*
HbA1c	−0.005	0.010	−0.057	−0.521	0.605
Age	−0.003	0.003	−0.119	−0.977	0.334
Weight	0.003	0.025	0.209	0.122	0.903
Height	0.001	0.022	0.037	0.036	0.971
BMI	0.000	0.066	−0.006	−0.004	0.997

*Indicates that the difference between groups is statistically significant (*p* < 0.05).

## 4 Discussion

Diabetes mellitus and osteoporosis are metabolic disorders sharing interrelated genetic susceptibilities and complex pathophysiological processes (18). T2DM directly influences bone strength and metabolism, with certain antidiabetic medications also exerting effects on skeletal homeostasis. These interactions contribute to T2DM-related complications, fall risks, and subsequent fracture likelihood (19, 20). T2DM patients exhibit higher trabecular bone density but lower cortical bone density, resulting in reduced bone strength (11). Akalin et al. (21) reported decreased bone formation marker levels in T2DM without significant changes in bone resorption markers, indicating slowed bone turnover. Abnormal glucose metabolism has numerous adverse effects on bone remodeling, such as osteopenia and increased fracture risk (22). For instance, persistent hyperglycemia and insulin resistance disrupt bone matrix metabolism and osteoblast differentiation via interfering with the PI3K/Akt signaling pathway and D-chiro-inositol (DCI)-related metabolic processes, thereby impairing bone matrix integrity and weakening metabolic regulatory capacity (23). Furthermore, some antidiabetic drugs can also impact bone metabolism. Thiazolidinediones (TZDs) activate PPARγ, inhibiting osteogenic differentiation and promoting adipogenesis, thereby reducing BMD and increasing fracture risk (24). In contrast, metformin suppresses osteoclast formation by reducing the expression of RANKL (receptor activator of nuclear factor kappa-B ligand) (25). Notably, both T2DM and osteoporosis independently contribute to cognitive impairment (26), with their concurrent presence potentially triggering a synergistic cascade effect.

Pathophysiological links exist between T2DM and cognitive impairment (27). Most T2DM patients are highly susceptible to various cognitive deficits, particularly dementia. T2DM patients with mild cognitive impairment exhibit significantly higher conversion rates to dementia compared to cognitively intact T2DM patients and non-diabetic MCI individuals (28). Evidence suggests an association between glucose regulation and cognitive impairment. In older adult T2DM patients, cognitive function is closely related to glycemic control and HbA1c levels, with cognitive performance declining as HbA1c increases (29). Xiao et al. reported a u-shaped association between HbA1c levels and cognitive impairment risk, particularly in global cognition and contextual memory, suggesting both excessively low and high HbA1c levels may be detrimental to cognitive function (30). However, our study found no correlation between HbA1c and cognitive performance, which we hypothesize relates to our data selection criteria. We enrolled a homogenous cohort of T2DM patients with minimal HbA1c variability, thereby isolating the effects of non-glucose-related factors on cognition while ensuring clinical homogeneity and controlling for confounding variables.

Osteoporosis, a prevalent comorbidity in diabetic individuals, demonstrates a multifactorial pathogenesis influenced by factors including diabetes subtype, disease duration, glucose-lowering agents, adiposity, fall propensity, sex, and chronological age. Following adjustment for glycemic perturbations, our investigation revealed statistically significant disparities in bone mineral density (BMD) between cognitively impaired and cognitively intact cohorts. Correlation analysis identified a positive correlation between age and BMD, corroborating findings from prior investigations (31). Bone metabolism has a significant age-related profile (32). In pediatric and adolescent populations, the skeletal system maintains anabolic metabolic equilibrium, characterized by augmented osteogenesis. Through adulthood, skeletal turnover remains relatively consistent, with osteoblastic activity and osteoclastic resorption maintaining dynamic parity. Around the sixth decade of life in females, both bone resorption and formation processes experience marked acceleration. However, this dual increase results in functional disequilibrium, as osteoclastic activity outpaces osteoblastic activity, leading to progressive reduction in bone mineral density. It should be noted that with increasing age, adipose tissue redistributes, with subcutaneous fat decreasing and visceral fat increasing (33). Visceral adipose tissue releases adipokines, thereby inducing hepatic secretion of acute-phase reactants (e.g., C-reactive protein, CRP) and promoting macrophage infiltration characterized by increased production of inflammatory cytokines (including IL-6, TNF-α, PAI-1, and MCP-1) (34). IL-6 can stimulate osteoclast activity, increasing bone resorption rates, while elevated circulating CRP levels are linked to higher levels of N-terminal telopeptide of type I collagen (NTx, a bone resorption marker) and lower bone mass (12). Therefore, scientifically guided weight management may benefit T2DM patients by improving both glycemic control and fracture risk prevention. Maintaining an optimal body composition could help mitigate inflammation-driven bone loss and support metabolic health in this high-risk population.

Renowned research teams have proposed the bidirectional regulation theory of the bone-brain axis (35), which closely links cognitive impairment and osteoporosis—two seemingly unrelated diseases—and has attracted extensive attention in the neuroscience field. Notably, as early as 2000, the Duchy team (36) first revealed the neural circuits and molecular mechanisms by which bone mass is regulated through the central nervous system. Their studies indicated that key regulators of bone formation are relayed via the hypothalamus to modulate osteogenesis, suggesting osteoporosis is at least partially a central nervous system disorder. Significantly, bone metabolism can also reciprocally regulate the central nervous system, with osteoporosis identified as an independent risk factor for early dementia development. Osteoporotic individuals exhibit a significantly higher dementia risk compared to healthy populations (37). Investigations in AD populations have identified the Wnt/β-catenin signaling pathway as a critical shared mechanism between AD and osteoporosis (38). This pathway promotes both bone formation and cerebral synaptogenesis while playing a core role in maintaining bone homeostasis. Specific deletion of β-catenin in osteocytes leads to significant reductions in cortical and trabecular bone mass, associated with enhanced osteoclastic bone resorption. Moreover, osteoclasts regulate bone remodeling by secreting Wnt ligands and chemokines that stimulate osteoblast differentiation (39). The Wnt/β-catenin signaling pathway also holds profound significance in AD pathogenesis. Activation of this pathway inhibits amyloid-β (Aβ) production and tau hyperphosphorylation in the brain, thereby influencing neuronal survival, neurogenesis, and the regulation of synaptic plasticity (40). Zhou et al. (41) found that women experiencing more rapid bone loss are more likely to undergo cognitive decline, with this association remaining statistically significant after adjusting for age, education, stroke history, functional impairment, and estrogen use. A community-based prospective cohort study further demonstrated the link between BMD and AD, revealing that women in the lowest quartile of femoral neck BMD had more than twice the risk of AD and dementia compared to other groups, with significant risk elevation persisting after multivariate adjustment (42). This association also applies to male populations (43). Thus, in the clinical management of T2DM patients, attention should be paid to both BMD measurement and cognitive assessment, which can improve treatment compliance and effectively prevent falls and fractures. Our analysis of subjects across different bone mass groups revealed significant differences in MoCA scores. Regression analysis indicated a significant positive correlation between BMD and MoCA scores, consistent with prior findings. However, Bradburn et al. (44) confirmed no association between total body bone mineral density and cognition in relatively healthy older adult individuals. During bone remodeling, various intermediate metabolites are generated, which may serve as critical factors in cognitive dysfunction. We posit that abnormal bone metabolism promotes cognitive impairment through correlational rather than causal relationships.

Interestingly, this study revealed significant differences in OC levels across groups stratified by either bone mass or cognitive changes, with positive correlations observed between OC content and both MoCA scores and BMD. OC, a non-collagenous, vitamin K-dependent bone matrix protein containing three γ-carboxyglutamic acid (GLA) motifs (45), not only promotes mineral deposition and bone remodeling (45, 46) but also modulates osteoclast and osteoclast precursor activity (46). Lu et al. (47) found that osteoporotic patients exhibit lower OC levels compared to individuals with normal BMD. A study (48) involving clinical patient samples and a type 2 diabetes rat model demonstrated a significant positive correlation between cognitive function and serum OC levels, with reduced OC levels in diabetic patients linked to insulin dysfunction. Insulin secretion is primarily regulated by the PI3K-AKT-GSK/3 signaling pathway, where GSK-3β serves as a key protein associated with both cognitive impairment and glycemic regulation (49). OC administration significantly increased phosphorylation levels of PI3K, AKT, and GSK-3β in the hippocampal tissue of diabetic rats, thereby achieving dual regulation of cognitive improvement and glucose homeostasis (48). Thus, decreased OC levels exacerbate insulin resistance, thereby influencing systemic glucose homeostasis and cognitive alterations. Additionally, OC can cross the blood-brain barrier (BBB) and participate in bone-brain axis communication via its receptors (50). Under chronic hyperglycemic conditions, hypermethylation of the Gpr158 gene promoter region in the hippocampus suppresses its expression, disrupting bone-brain axis balance and impairing brain function (51). Additionally, OC binds to brainstem and midbrain neurons through mechanisms such as enhancing monoamine neurotransmitter synthesis and inhibiting GABA production. Independent of its classical metabolic functions, reduced OC levels can result in anxiety, depression, and impaired learning and memory abilities (50). In summary, investigating the mechanistic roles of OC in glucose regulation and cognitive impairment could facilitate the development of novel therapeutic strategies for diabetes and identify potential therapeutic targets.

## 5 Conclusion

T2DM demonstrates intricate pathophysiological connections with osteoporosis and cognitive dysfunction, involving core mechanisms such as metabolic homeostasis perturbation, chronic low-grade inflammation, and disruption of the bidirectional bone-brain axis regulatory network. OC, a pivotal osteogenic mediator linking bone metabolism, glycemic homeostasis, and neurocognitive function, not only maintains bone remodeling equilibrium through modulating osteoblast-osteoclast coupling but also traverses the BBB via endocrine pathways to participate in synaptic plasticity regulation and insulin signaling cascade within the central nervous system. Elucidating the cross-impact of T2DM-related metabolic derangements on the bone metabolism-neurocognitive axis not only facilitates the development of early screening models integrating BMD monitoring and cognitive function assessment but also provides theoretical frameworks for designing personalized therapeutic strategies. Multidisciplinary collaboration among endocrinologists, orthopedists, and neurologists can significantly enhance the holistic prognosis of T2DM patients.

## Data Availability

The raw data supporting the conclusions of this article will be made available by the authors, without undue reservation.
